# Preparation and characterization of (−)-Epigallocatechin-3-gallate (EGCG)-loaded nanoparticles and their inhibitory effects on Human breast cancer MCF-7 cells

**DOI:** 10.1038/srep45521

**Published:** 2017-03-28

**Authors:** Liang Zeng, Jingna Yan, Liyong Luo, Mengjun Ma, Huiqun Zhu

**Affiliations:** 1 College of Food Science, Southwest University, Beibei, Chongqing, 400716, China; 2 Tea Research Institute, Southwest University, Beibei, Chongqing, 400716, China; 3 Xianning Agriculture Academy of Sciences, Xianning, Hubei, 437100, China; 4 Institute of Architecture and Design, Chongqing College of Humanities Science & Technology, Hechuan, Chongqing, 401524, China

## Abstract

We were employing nanotechnology to improve the targeting ability of (−)-Epigallocatechin-3-gallate (EGCG) towards MCF-7 cells, and two kinds of EGCG nanoparticles (FA-NPS-PEG and FA-PEG-NPS) were obtained, besides, their characteristics and effects on MCF-7 cells were studied. The results indicated that (i) both FA-NPS-PEG and FA-PEG-NPS have high stabilities; (ii) their particles sizes were 185.0 ± 13.5 nm and 142.7 ± 7.2 nm, respectively; (iii) their encapsulation efficiencies of EGCG were 90.36 ± 2.20% and 39.79 ± 7.54%, respectively. (iv) there was no cytotoxicity observed in EGCG, FA-NPS-PEG and FA-PEG-NPS toward MCF-7 cells over all concentrations (0~400 μg/mL) tested; (v) EGCG, FA-NPS-PEG and FA-PEG-NPS inhibited MCF-7 cells proliferation in dose-dependent manners, with the average IC_50_ of 470.5 ± 33.0, 65.9 ± 0.4 and 66.6 ± 0.6 μg/mL; (vi) EGCG, FA-NPS-PEG and FA-PEG-NPS could modulated the expressions of several key regulatory proteins in PI3K-Akt pathway such as up-regulation of PTEN, p21 and Bax, and down-regulation of *p*-PDK1, *p*-AKT, CyclinD1 and Bcl-2, which gave an illustration about the mechanism by which EGCG nanoparticles inhibited MCF-7 cells proliferation. In this study, EGCG nanoparticles can significantly enhance the targeting ability and efficacy of EGCG, which is considered to an experimental foundation for further research on its activity, targeting ability and metabolism *in vivo*.

Cancer has been the second leading cause of death ranked after heart disease^1^. Among all types of cancers, breast cancer is the second leading cause of death and has been estimated to account for 29% of new cancer cases in females^2^. While in females of United States especially those aged from 20 to 49, breast cancer becomes the first leading cause of death^2^. Considering the present case amount and its growing potential, breast cancer has become a greatest threat to women’s health. Although there is no wonder drug for curing cancer at present, it has been estimated that more than two thirds of human cancers could be possibly prevented by appropriate lifestyle modifications including dietary modification^3^. Previous studies have demonstrated that regular consumption of whole grains, fruits and vegetables is negatively correlated with the risk of developing many types of chronic diseases including cardiovascular disease, type 2 diabetes and some cancers^4^,^5^,^6^. Various nutrients and phytochemicals such as phenolics, carotenoids, vitamins, minerals and fibers contained in natural products were believed to achieve their major health benefits^7^. Moreover, a number of anticancer drugs such as phenolic compounds, homoharingtonine and arsenic trioxide currently used in the clinic are of natural origin and so are the anticancer drugs approved internationally in the past few decades^8^,^9^. Due to the application potential in anticancer, phytochemicals, which are defined as bioactive nonessential nutrients derived from plants, have become a promising focus in anticancer research.

(−)-Epigallocatechin-3-gallate (EGCG) is a rich phytochemical resource contained in tea, accounting for 18~36% of total phenolics and 70% of total catechins^10^. Due to its favorable biological properties, EGCG has received considerable attention as a potential treatment for a variety of diseases, including cancers^11^,^12^. Experimental studies both in animal models and cell lines have consistently demonstrated that EGCG strongly induced apoptosis and growth inhibition in several types of human cancers, such as brain, kidney, leukemia, colon and breast^3^. However, the *in vivo* studies about the efficacy of EGCG were not always consistent with the *in vitro* studies, since the effects of EGCG on multiple types of cancers were sometimes disappointing *in vivo* trails, which may be attributed to the low bioavailability and weak targeting ability of EGCG^13^,^14^. To improve the targeting ability of chemo preventive agents, folic acid (FA) has been extensively employed as a modifier in the development of nanoparticles for cancer treatment, since FA has highly selectivity for recognizing folate receptors (FR), which are known to be overexpressed on the surface of various cancer cells, including ovarian, lung, breast, endometrial, renal, and colon cancers^15^,^16^. Yuan-gang Zu *et al*.^17^ have demonstrated that FA mediated EGCG bovine serum albumin nanoparticles (FA-EGCG-BSANP) significantly promote EGCG to PC-3 cells sites and improve their efficacy. Recently, nanotechnology was introduced and employed to improve the bioavailability of chemo preventive agents, such as EGCG^18^,^19^. And CS nanoparticles encapsulating EGCG has received considerable attention for several types of cancers prevention and therapy, since previous study has been proposed that CS has many significant biological and chemical properties such as low toxicity, biodegradability, biocompatibility, fungistatic, hemostatic, bacteriostatic, and anti-carcinogen properties^20^, and that CS particles could protect functional components against pH and enzymatic degradation in the gastrointestinal tract to achieve a higher oral bioavailability of drugs^11^. Additionally, polyethylene glycol (PEG) has also been extensively employed as a modifier, since PEG was reported to be able to improve the water solubility of nanoparticles and prevent absorption of plasma proteins, thereby prolonging nanoparticles blood circulation time and allowing the nanoparticles to reach their target tissues^15^,^21^.

Despite the efforts that devoted to confirm inhibition effect of EGCG on human breast cancer MCF-7 cells, more works have been done to examine the mechanism by which EGCG inhibit MCF-7 cells proliferation. Luo *et al*. have elucidated the mechanism by investigating the effects of EGCG on the expression of hypoxia-inducible factor 1α (HIF-1α) and vascular endothelial growth factor (VEGF), and demonstrated that protein expression of HIF-1α and VEGF declined in a dose-dependent manner in MCF-7 cells pretreated with increasing concentrations of EGCG, further induced the decrease of cell growth^22^. In addition, EGCG exerted its anti-proliferation effect by the downregulation of estrogen receptor alpha (ERα) function^23^,^24^, modulation of the PI3K-Akt pathway and Bcl-2 family proteins^25^, inhibition of cyclin-dependent kinases 2 (Cdk2) and 4 (Cdk4) and induction of Cdk Inhibitors p21 and p27^26^. With regard to all the mechanisms researched, the abnormal activation of the PI3K-Akt pathway, one of the major downstream mediators of increased mitogenic signaling, has been validated by epidemiological and experimental studies as an essential step toward the initiation and maintenance of human tumors^27^,^28^,^29^. And blocking the PI3K-Akt pathway was reported to simultaneously inhibit the proliferation and growth of tumor cells and sensitize them toward programmed cell death^27^, making this pathway an attractive target for therapeutic strategies to treat various types of cancer^28^.

In this paper, inspired by the theory of nanoparticle-mediated deliver system, EGCG nanoparticles were prepared through encapsulating EGCG in CS-TPP nanoparticles, and modified by FA and PEG, and characterized by Fourier transform infrared (FTIR) spectroscopy, Nuclear magnetic resonance (NMR) spectroscopy, Zetasizer Nano-ZS90, Scanning electron microscope (SEM) and High performance liquid chromatography (HPLC) to confirm their structures, stabilities, particle sizes, morphologies and encapsulation efficiency (EE) of EGCG respectively. Moreover, the effects of EGCG and EGCG nanoparticles on morphology, growth, and proliferation of MCF-7 cells were evaluated and their targeting ability to MCF-7 cells was observed using laser scanning confocal microscope and fluorophotometer microscope. Finally, the mechanisms by which EGCG and EGCG nanoparticles inhibited MCF-7 cells proliferation were illustrated through examining theireffects of EGCG nanoparticles on several key regulatory proteins in PI3K-Akt pathway in MCF-7 cells.

## Results

### Synthesis of EGCG nanoparticles

**Figure 1:**
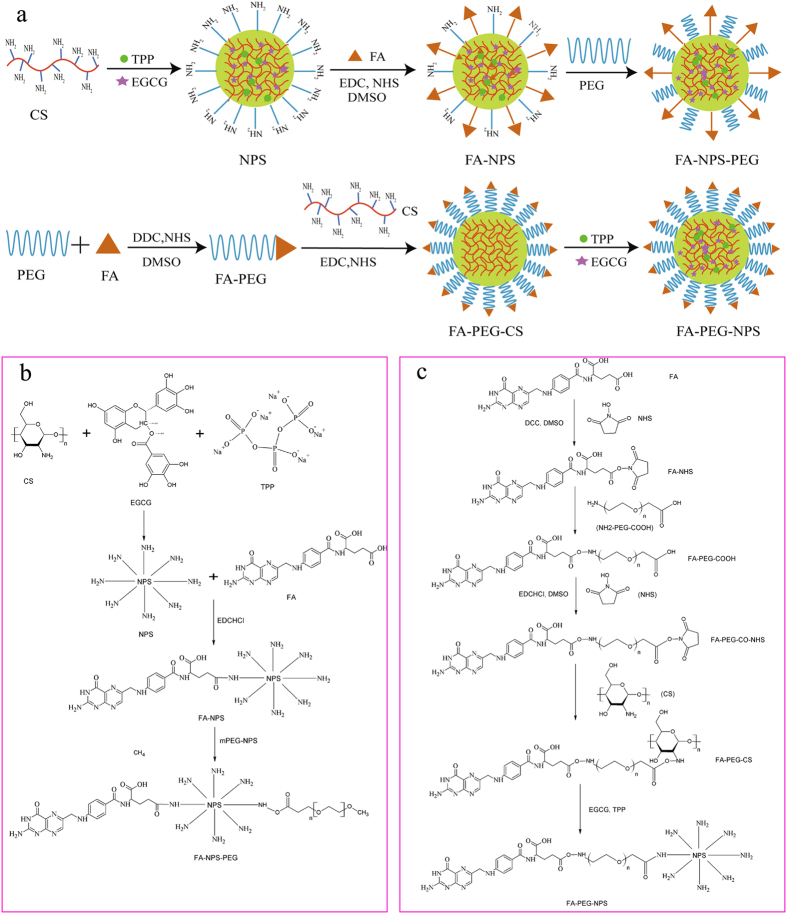
Synthesis pathways of FA-NPS-PEG and FA-PEG-NPS. (**a**) Intuitive diagram for synthesis of FA-NPS-PEG and FA-PEG-NPS. (**b**) Chemical reaction scheme for synthesis of FA-NPS-PEG. (**c**) Chemical reaction scheme for synthesis of FA-PEG-NPS.

Aiming at enhancing the targeting ability and anti-proliferative activity of EGCG on MCF-7 cells, EGCG nanoparticles modified by FA and PEG were prepared through two different ways^11^,^15^,^30^, and finally two kinds of EGCG nanoparticles were obtained, namely FA-NPS-PEG and FA-PEG-NPS (Fig. 1a); meanwhile, another three kinds of EGCG nanoparticles modified by FA or PEG alone, namely CS-EGCG-TPP nanoparticles (NPS), PEG-NPS and FA-NPS, were obtained during the preparation of FA-NPS-PEG. Therefore, these five kinds of EGCG nanoparticles would be used for further measurement.

During the experiment, FA-NPS-PEG was prepared with a three steps procedure as depicted in Fig. 1b. Firstly, NPS were prepared based on ionotropic gelation technique between positively charged amine groups (-NH_2_) on CS and negatively charged tripolyphosphate groups on TPP, and EGCG was attached to CS via covalent bonding and hydrogen bonding^31^. Secondly, the carboxylic groups (-COOH) of FA was activated using 1-(3-Dimethylaminopropyl)-3-ethylcarbodiimide hydrochloride (EDC·HCl), and then grafted FA with NPS through an amide (-CONH-) formation reaction between activated -COOH groups of FA and -NH_2_ groups of CS, forming FA-NPS^15^,^30^,^32^. Thirdly, PEG was conjugated to FA-NPS via -CONH- bond formation reaction between ester group (-COOR) of succinimidyl ester of PEG propionic acid (mPEG-SPA) and -NH_2_ group of CS, forming FA-NPS-PEG^21^. Moreover, PEG-NPS was synthesized by grafting PEG onto NPS via -CONH- bond formation reaction between -COOR group of mPEG-SPA and -NH_2_ group of CS.

FA-PEG-NPS was prepared with a five steps procedure as depicted in Fig. 1c. Firstly, the -COOH groups of FA was activated with a mixture of *N*-Hydroxysuccinimide (NHS), dicyclohexylcarbodiimide (DCC) and anhydrous dimethyl sulfoxide (DMSO) and converted to reactive intermediate FA-NHS (γ)^21^. Secondly, FA was attached to hetero-functional PEG derivative (NH_2_-PEG-COOH) via the well-known carbodiimide chemistry to obtain FA-PEG-COOH^33^. Thirdly, the -COOH groups of FA-PEG-COOH was again activated with the mixture of EDC·HCl, NHS and DMSO to convert to the reactive intermediate FA-PEG-CO-NHS^33^,^34^. Fourthly, FA-PEG-CO-NHS were subsequently grafted onto CS to achieve the FA-PEG-CS conjugate^33^,^34^. Finally, FA-PEG-NPS was prepared in the same way as NPS.

### Characterization of EGCG nanoparticles

**Figure 2:**
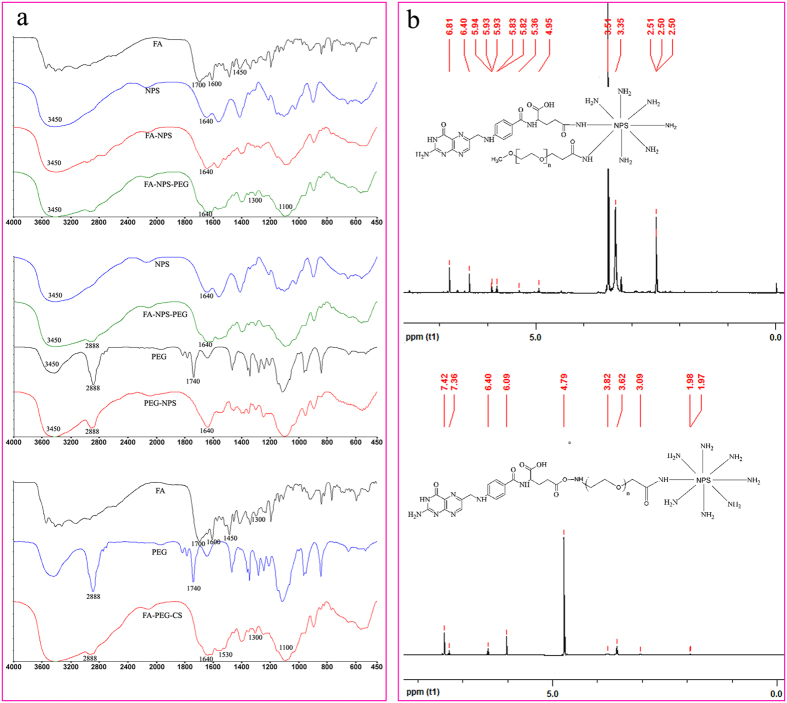
FTIR spectra (**a**) and ^1^H-NMR spectra (**b**) of FA-NPS-PEG and FA-PEG-NPS.

The resulting EGCG nanoparticles conjugated with PEG and/or FA were characterized by FTIR to confirm the covalent binding of both PEG and FA to the surface of NPS^15^, as shown in Fig. 2a. In the spectra of FA-NPS-PEG and FA-NPS, the characteristic peak at 3450 cm^−1^ was attributed to the –NH_2_ and –OH groups on CS and FA, and the characteristic peak at 1100 cm^−1^ represented C-O stretch on CS. The appearance of characteristic peak at 1300 cm^−1^ (belongs to C=N bond on FA) indicated the successful attachment of FA onto CS. On the other hands, the characteristic peak (1700 cm^−1^) representing -COOH groups on FA was made to disappeared, and a high intensity peak at 1640 cm^−1^ appeared, which corresponded to a classical -CONH- bond formation between FA and CS, also indicating the successful attachment of FA onto CS. Compared the spectrum of FA-NPS-PEG with that of PEG-NPS, the appearance of characteristic peak at 2888 cm^−1^ (belongs to C-H stretch on PEG) and the disappearance of characteristic peak at 1740 cm^−1^ indicated the successful attachment of PEG onto CS, meanwhile, PEG maybe has made a change. In the spectrum of FA-PEG-CS synthesized during the preparation of FA-PEG-NPS, the characteristic peaks at 1100 cm^−1^, 1300 cm^−1^, 1530 cm^−1^ and 2888 cm^−1^ were attributed to C-O stretch on CS, C=N stretch on FA, –NH groups on CS and C-H stretch on PEG, respectively. And the characteristic peaks at 1700 cm^−1^ (belongs to –COOH group on FA) and 1740 cm^−1^ (belongs to -COOH on NH_2_-PEG-COOH) were disappeared and the characteristic peak at 1640 cm^−1^ became stronger, which corresponded to NH_2_ on CS and classical -CONH- bonds formation between FA and PEG, PEG and CS. All of these indicated that FA-PEG-CS was synthesized successfully.

Concurrently, successful synthesis of FA-NPS-PEG and FA-PEG-NPS were also confirmed by ^1^H NMR method, the most effective technique that is always used to determine the structure of CS derivatives^30^, as shown in Fig. 2b. From the typical ^1^H NMR spectrum of FA-NPS-PEG, the signals at 3.35 and 3.51 ppm corresponding to –CH_2_CH_2_O- and –OCH_3_ of mPEG-SPA^21^,^30^, respectively, indicated that PEG has been successfully conjugated with NPS. The signals at 4.95–5.94 ppm revealed the presence of CS. Coupling of FA residue successfully grafted NPS was confirmed by the appearance of signals at 6.40–6.81 ppm corresponding to the aromatic protons of FA. In the ^1^H NMR spectrum of FA-PEG-CS, the signals at 1.97–1.98 and 3.09 ppm were attributed to acetyl group (-COCH_3_) and monosaccharide residue (CH-NH-) of CS^21^,^32^. The bond between CS and PEG corresponding to the signal of –NH-CH_2_CH_2_O- appeared at 3.62 ppm^32^,^35^. The presence of PEG group was also confirmed from the signals at 3.82 ppm corresponding to CH_2_CH_2_O of PEG^21^, and the presence of FA group was confirmed by the appearance of signals at 6.09–7.42 ppm, which corresponded to the aromatic protons of FA^32^.

**Figure 3:**
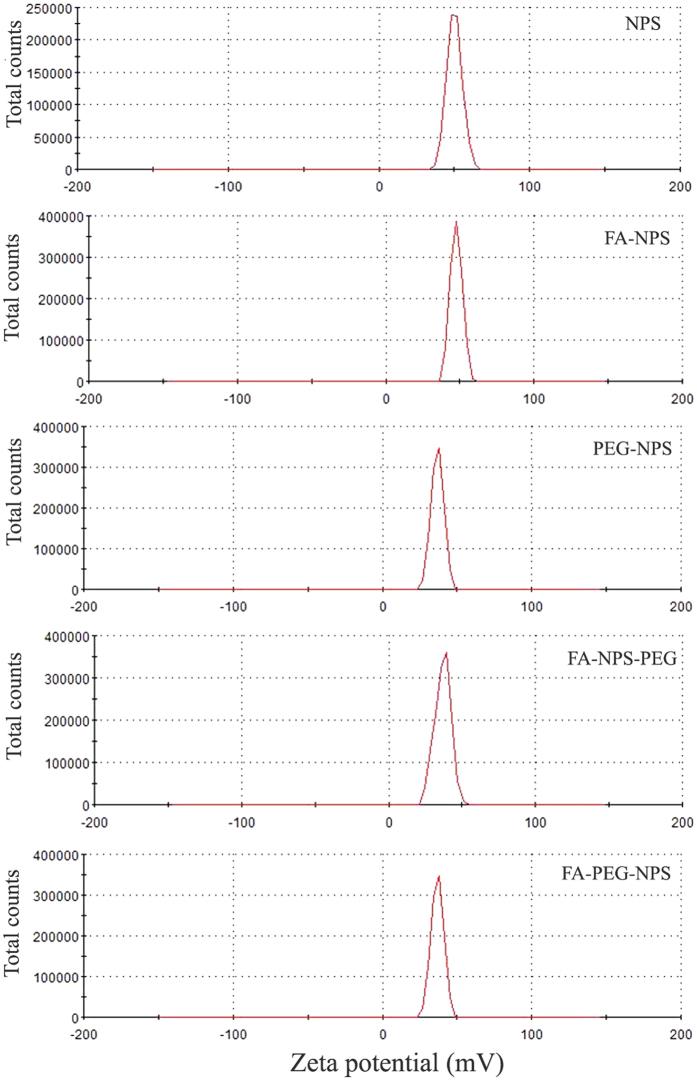
The zeta potentials of NPS, FA-NPS, PEG-NPS, FA-NPS-PEG and FA-PEG-NPS.

Zeta potential is another key parameter reflecting the density of the surface charge, which contributed to the adhesion properties and transport properties of the nanoparticles^36^. High zeta potential generally indicates high stability of nanoparticles and the minimum zeta potential required for a physical stable nano-suspension is ±30 mV^37^,^38^. The zeta potentials of NPS, FA-NPS, PEG-NPS, FA-NPS-PEG and FA-PEG-NPS at same pH were shown in Fig. 3. Compared the zeta potentials of PEG-NPS, FA-NPS-PEG, FA-PEG-NPS with those of NPS, FA-NPS, we found that the zeta potentials of EGCG nanoparticles decreased when conjugated with PEG, which may be due to that the molecular chain of PEG doesn’t have any ionizable groups^39^. On the other hands, the change of zeta potentials also suggested the successful attachment of PEG onto NPS. As shown in Fig. 3, all of the zeta potentials values of EGCG nanoparticles were higher than +30 mV, indicating high stability of five kinds of EGCG nanoparticles.

**Figure 4:**
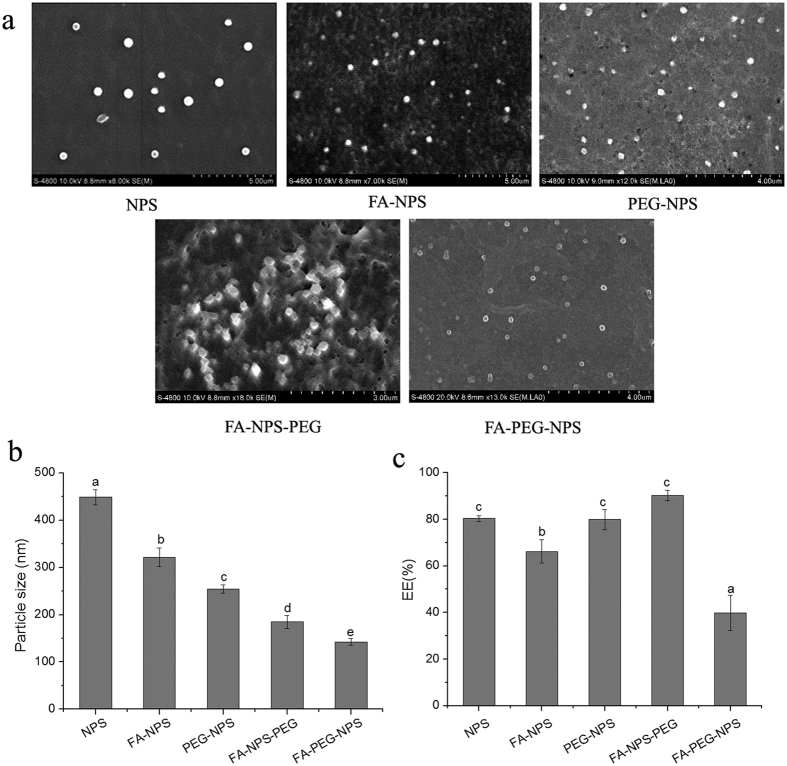
Characteristics of synthesized NPS, FA-PEG, PEG-NPS, FA-NPS-PEG and FA-PEG-NPS. (**a**) SEM image. (**b**) Particle sizes. (**c**) EEs of EGCG. Different letters on the column of graphs indicates a significant difference from the control. (*p* < 0.05).

The morphological characteristics of five kinds of EGCG nanoparticles were visualized by SEM and presented in Fig. 4a; besides, the particles sizes of EGCG nanoparticles obtained from SEM were also presented in Fig. 4b. From Fig. 4a well defined spherical shapes were obtained for all of EGCG nanoparticles, which were due to ionotropic gelation technique between CS and TPP^31^, and modification by PEG and/or FA seemed to have no significant effect on the morphological characteristics of EGCG nanoparticles. Both Fig. 4a and b presented that the particles sizes of five kinds of EGCG nanoparticles followed in an order: FA-PEG-NPS＜FA-NPS-PEG＜PEG-NPS＜FA-NPS＜NPS. Compared NPS with other EGCG nanoparticles, it was very desirable to note that EGCG nanoparticles had smaller particle sizes after conjugated with FA and/or PEG. The comparison of particle sizes between PEG-NPS (254.8 ± 9.1 nm) and FA-NPS (321.4 ± 20.3 nm) revealed that PEG was more effective in decreasing the particle size of EGCG nanoparticles compared to FA. Moreover, EGCG nanoparticles conjugated with PEG and FA, those were FA-NPS-PEG and FA-PEG-NPS, had smaller particle sizes than FA-NPS and PEG-NPS conjugated with either FA or PEG alone, indicating synergistic effects of PEG and FA was more effective than individual effects. Although both FA-NPS-PEG (185.0 ± 13.5 nm) and FA-PEG-NPS (142.7 ± 7.2 nm) conjugated with PEG and FA, they didn’t have equal particles sizes, revealing that the way of preparing had an effect on the particle size of EGCG nanoparticles.

EE of EGCG is defined as percentage of EGCG loading content that can be entrapped into EGCG nanoparticles^36^. As shown in Fig. 4c, the EEs of EGCG in five kinds of EGCG nanoparticles followed in an order: FA-PEG-NPS＜FA-NPS＜PEG-NPS＜NPS＜ FA-NPS-PEG, revealing that modification by PEG and/or FA had an effect on the EE of EGCG in EGCG nanoparticles, which may contributed to that the modification had changed the structure of hydrogen bond of EGCG nanoparticles. During the experiment, both FA-NPS-PEG and FA-PEG-NPS were modified by PEG and FA, however, the EE of FA-NPS-PEG (90.36 ± 2.20%) was highest, while the EE of FA-PEG-NPS (39.79 ± 7.54%) was lowest, indicating that the way of preparing also can influence the EE of EGCG in EGCG nanoparticles. Compared the EEs of EGCG in FA-NPS and PEG-NPS with that in NPS, the EE of EGCG in EGCG nanoparticles decreased after modified by either PEG or FA alone. Therefore, to achieve higher EE of EGCG, NPS should be prepared first, followed by modification with PEG and FA.

### Effects of EGCG nanoparticles on cellular morphology of MCF-7 cells

**Figure 5:**
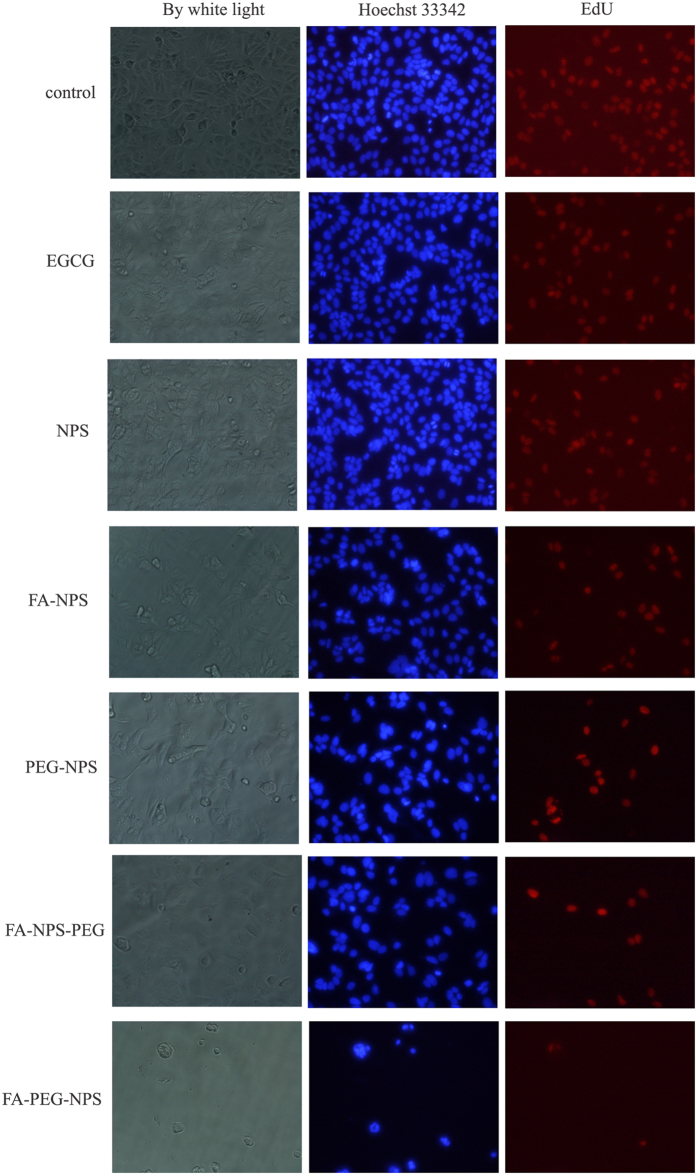
Morphology of MCF-7 cells treated with EGCG and synthesized EGCG nanoparticles. By white light (gray), Hoechst 33342 (Blue), and EdU (red).

MCF-7 cells were incubated with five kinds of EGCG nanoparticles, respectively, and processed for 5-Ethynyl-2′-deoxyuridine (EdU) and Hoechst 33342 staining, followed by observation using an inverted fluorescence microscope. EdU was used to label dividing cells, detected with a fluorescent azide^40^, while Hoechst 33342 was used to labeling DNA by binding to the minor groove of the DNA^41^,^42^. Representative staining images are shown in Fig. 5, in which EdU-labelled cells were labeled red and Hoechst 33342-labelled cells were labeled blue. The attachment of polygonal-shaped cells to 96-well plate was observed in control group, achieving 80~90% confluence. Compared with the control group, cells were treated with EGCG (200 μg/mL) or EGCG nanoparticles (loading 200 μg/mL of EGCG) for 48 h, which changed the cell morphology from a polygonal shape to a circular shape. Concurrently, both the total number of MCF-7 cells and the number of cells in proliferation period significantly decreased compared to the control group. It was noted that EGCG nanoparticles modified by either FA or PEG could greatly reduce the number of adherent cells, again indicating that FA or PEG had been successfully conjugated with EGCG nanoparticles and perfectly played their target roles. Fewer MCF-7 cells were obtained when treated with FA-NPS-PEG and FA-PEG-NPS compared to the others, especially FA-PEG-NPS, revealing that synergistic modification of PEG and FA was more effective than individual modification, and that FA-PEG-NPS had the best effect on decreasing the number of MCF-7 cells, which may be attributed to the way of preparing.

### Effects of EGCG nanoparticles on proliferation of MCF-7 cells

**Figure 6:**
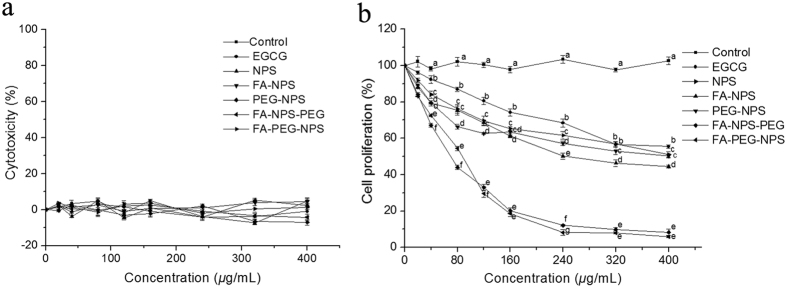
Effect of EGCG and synthesized EGCG nanoparticles on cytotoxicity (**a**) and cell proliferation rates (**b**) of MCF-7 cells. Different letters under the same concentration indicates a significant difference from the control. (*p* < 0.05).

To establish that EGCG in EGCG nanoparticles retained its mechanistic identity, the cytotoxicity and anti-proliferative activities of EGCG has been evaluated in this study. The cytotoxicity of EGCG and EGCG nanoparticles towards to the growth of MCF-7 cells was presented in Fig. 6a. The result showed that there was no cytotoxicity observed in EGCG and EGCG nanoparticles toward MCF-7 cells over all concentrations tested. The cell proliferation rates of MCF-7 cells treated with or without EGCG and EGCG nanoparticles were presented in Fig. 6b. Compared to the control group, the cell proliferation rates of MCF-7 cells decreased significantly in a dose-dependent manner. And the average IC_50_ calculated using CalcuSyn Demo software were 470.5 ± 33.0, 410.8 ± 32.5, 286.4 ± 20.7, 343.6 ± 18.8, 65.9 ± 0.4 and 66.6 ± 0.6 μg/mL for EGCG, NPS, FA-NPS, PEG-NPS, FA-NPS-PEG and FA-PEG-NPS, respectively, indicating that the anti-proliferative activities followed in an order: FA-NPS-PEG>FA-PEG-NPS>FA-NPS>PEG-NPS>NPS>EGCG. It was very interesting to note that the cell proliferation rates of FA-NPS-PEG and FA-PEG-NPS didn’t show significant difference at the concentrations of EGCG ranged from 0 to 400 μg/mL (Fig. 6b), indicating their equal antiproliferative activities, which were in consistent with the average IC_50_ values. When MCF-7 cells treated with FA-NPS-PEG and FA-PEG-NPS loading 400 μg/mL EGCG, the cell proliferation rates reduced to 91.71% and 94.14%, respectively, suggesting that MCF-7 cells proliferation were almost completely inhibited.

### Cellular uptake of EGCG nanoparticles

**Figure 7:**
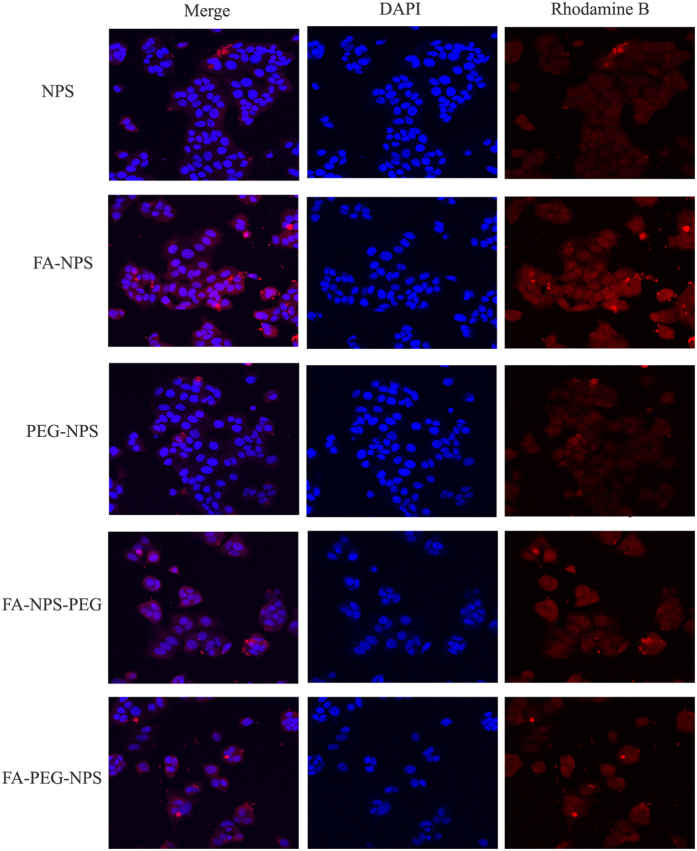
Confocal images of MCF-7 cells incubated with synthesized EGCG nanoparticles for 24 h. DAPI-labelled nuclei (blue) and Rhodamine B-labelled EGCG nanoparticles (red).

In order to establish the targeting ability of EGCG nanoparticles, cellular uptake of Rhodamine B isothiocyanate-labeled EGCG nanoparticles by MCF-7 cells were visualized by laser scanning confocal microscope, as shown in Fig. 7. As seen, nearly no nanoparticles uptake was observed in NPS and PEG-NPS groups, as evidenced by the lack of any red fluorescence associated with cells. However, MCF-7 cells cultured with FA-NPS, FA-NPS-PEG and FA-PEG-NPS had shown considerably higher nanoparticles uptake than those cultured NPS and PEG-NPS, which may due to the presence of FA in EGCG nanoparticles which has good binding capacity to the cells having FR^43^. From Fig. 7 it was noted that the decreases in the numbers of MCF-7 cells cultured with FA-NPS-PEG and FA-PEG-NPS were clearly visible compared to the other three groups, indicating the more effective antiproliferative activities of FA-NPS-PEG and FA-PEG-NPS once more.

### Effects of EGCG nanoparticles on expressions of several key regulatory proteins in PI3K-Akt pathway in MCF-7 cells

**Figure 8:**
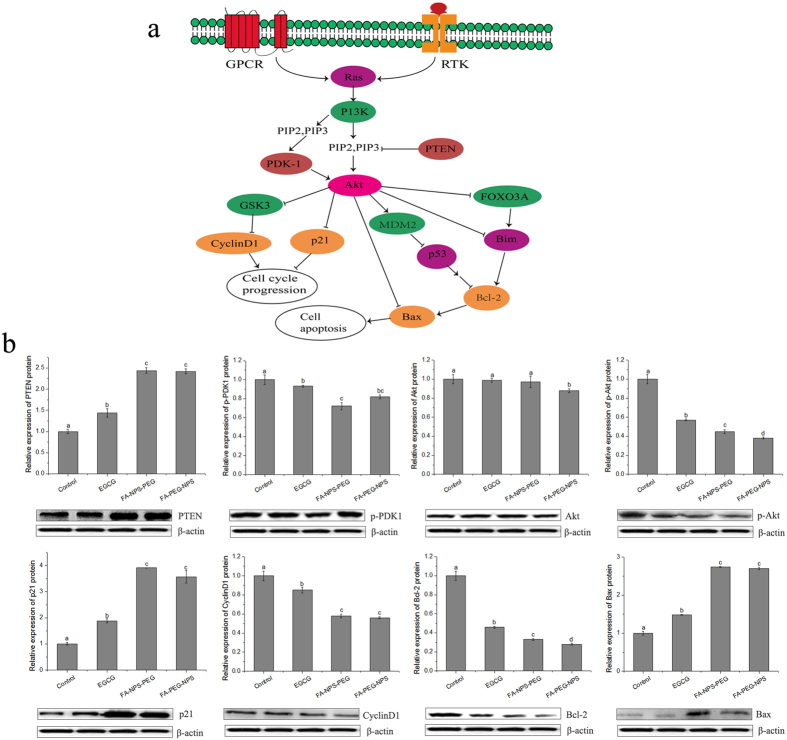
Overview of several key regulatory proteins in PI3K-AKT pathway in MCF-7 cells (**a**) and their expressions under the modulation of EGCG, FA-NPS-PEG and FA-PEG-NPS (**b**). *β*-actin was used as an internal control. Arrowheads indicate stimulation of downstream substrates; perpendicular lines indicate inhibition of downstream substrates. Different letters on the column of graphs indicates a significant difference from the control (*p* < 0.05).

In order to examined the mechanism by which EGCG and EGCG nanoparticles inhibited MCF-7 cells proliferation, several key regulatory proteins in PI3K-Akt pathway (Fig. 8a) were derived from MCF-7 cells pretreated with EGCG or EGCG nanoparticles and measured with western blot analysis, which is a powerful method for detecting target proteins on a nitrocellulose or polyvinylidene fluoride (PVDF) membrane^44^. With respect to EGCG or EGCG nanoparticles, western blot analysis showed a marked decrease of phosphotidylinositol-dependent kinase-1 (*p*-PDK1), *p*-Akt, CyclinD1 and Bcl-2, and a tendency toward higher levels of phosphatase and tensin homologue deleted on chromosome 10 (PTEN), p21 and Bax in MCF-7 cells compared to the control group, as shown in Fig. 8b. The comparison of proteins levels between EGCG and EGCG nanoparticles revealed that EGCG nanoparticles were superior to EGCG with regard to modulation of the PI3K-Akt pathway, which consisted with the preceding anti-proliferative activities. From Fig. 8b most of the proteins levels in FA-NPS-PEG group showed slight difference from that in FA-PEG-NPS group, suggesting little significant difference was shown in their abilities to modulate PI3K-Akt pathway. Together, these results suggested that EGCG and EGCG nanoparticles exerted their growth-inhibitory effects through modulation of the activities of several key regulatory proteins in PI3K-Akt pathway such as up-regulation of PTEN, p21 and Bax, and down-regulation of *p*-PDK1, *p*-Akt, Cyclin D1 and Bcl-2.

## Discussion

During this experiment, aiming at enhancing the targeting ability of EGCG towards MCF-7 cells, EGCG nanoparticles, namely FA-NPS-PEG and FA-PEG-NPS, were prepared on the basis of ionotropic gelation technique between CS and TPP and modified by FA and PEG. These results suggested that both FA-NPS-PEG and FA-PEG-NPS have been successfully synthesized and modified by FA and PEG, which were confirmed using FTIR and ^1^H NMR; and that both of the two EGCG nanoparticles possessed high stability and small particles sizes, which may provide a possibility for higher intracellular uptake^45^. However, with regard to the EEs of EGCG, FA-NPS-PEG retained 90.36 ± 2.20% of total EGCG, more than 2-fold higher than FA-PEG-NPS (39.79 ± 7.54%), which may due to the different way preparing the two EGCG nanoparticles. It seemed that the way preparing FA-NPS-PEG was more promising in improving the targeting ability of EGCG compared to that preparing FA-PEG-NPS.

Consistent with previous literatures that focused on the relationship between EGCG treatment and cancers prevention^12^,^46^, our results also demonstrated that EGCG and EGCG nanoparticles suppressed the proliferation of MCF-7 cells in a dose-dependent manner and that the effectiveness of EGCG nanoparticles, namely FA-NPS-PEG and FA-PEG-NPS, were superior to that of EGCG. Further, the observations of cellular morphology and cellular uptake studies have also illustrated the anti-proliferative activities of EGCG and EGCG nanoparticles, as evidenced by decreases in the numbers of MCF-7 cells. From cell proliferation studies, cellular uptake studies and western blot analysis, no significant difference in efficacies were observed between FA-NPS-PEG and FA-PEG-NPS, both of which were superior to EGCG. Considering the smaller particles sizes, lower EE of EGCG and nearly the equal efficacy of FA-PEG-NPS compared to that of FA-NPS-PEG, FA-PEG-NPS was supposed to be more effective with respect to the target abilities and inhibition effect on proliferation of MCF-7 cells. However, further study is still needed to improve the EE of EGCG in FA-PEG-NPS to achieve greater cancer chemoprevention effects of EGCG.

In order to illustrate the mechanism by which EGCG and EGCG nanoparticles inhibited MCF-7 cells proliferation, we investigated the effects of EGCG and EGCG nanoparticles on the expressions of several key regulatory proteins in PI3K-Akt pathway, including PTEN, p21, Bax, *p*-PDK1, *p*-Akt, Akt, Cyclin D1 and Bcl-2. Previously, plenty of efforts have been done to develop new inhibitors of the PI3K-Akt pathway, which always target key regulatory proteins such as PTEN^29^, PDK1^47^, Akt^48^ or PI3K^49^, since increasing evidence has been validated that blocking the PI3K-Akt pathway could simultaneously inhibit the proliferation and growth of tumor cells and sensitize them toward programmed cell death^27^. Both *in vitro* and *in vivo* studies have found that wortmannin have high selectivity for inhibiting PI3K, which was exerted by binding to the p110 catalytic subunit^49^, but the dose of wortmannin employed should be examined because of its inhibition of other enzymes at the same time^49^. LY294002, a competitive and reversible inhibitor of the ATP binding site of PI3K, has been shown to cause a G1 phase cell cycle arrest and inhibit Akt phosphorylation and activity and induce apoptosis in cells^49^,^50^. In consistent with previous studies^25^,^26^,^51^,^52^, our results also showed that EGCG potently inhibit cell proliferation and suppress tumor growth *in vitro* through inhibiting the activition of PI3K-Akt pathway that, in turns, resulting in alternations of key regulatory proteins levels. Compared to wortmannin or LY294002, which have several disadvantages such as difficulties with stability, solubility and toxicity that may limit their use in clinical trials^49^,^53^, EGCG is more attractive as an inhibitor of PI3K-Akt pathway because of its low toxicity, natural compounds, less cost, aqueous solubility, easy accessibility and daily consumption^54^. Besides, both FA-NPS-PEG and FA-PEG-NPS prepared in this study showed better abilities in altering the expressions of key regulatory proteins in PI3K-Akt pathway compared to EGCG, suggesting that nano-technology and modification with PEG and FA could enhance the effectiveness of EGCG treatments. Moreover, it was desirable to note that combined treatment, such as wortmannin and radiation^55^, wortmannin and paclitaxel^56^, paclitaxel and LY294002^56^, greatly potentiated apoptosis compared to either treatment alone. But whether the efficacy of EGCG or EGCG nanoparticles in inhibiting tumor growth will be further enhanced by combining EGCG or EGCG nanoparticles with radiation or existing chemotherapeutic agents awaits further study.

Previous studies has demonstrated that the abnormal activation of PI3K-Akt pathway can result from dysregulations of several components in this signaling system, including overexpression of the downstream kinase Akt, amplication of PI3K, presence of activating mutations in the PIK3CA, mutational inactivation of the tumor suppressor gene PTEN, and so on^27^,^28^. The serine/threonine kinase Akt, also called protein kinase B or PKB, is the most important downstream effector of PI3K and has been proved to play an important role in tumorigenesis and tumor growth^49^, which can be due to that Akt protects tumor cells from death through phosphorylation and inactivation of downstream substrates^27^,^29^, such as antiapoptotic proteins Bcl-2, proapoptotic proteins Bax and Bad^57^, ASK-1, cyclin-dependent kinase p21 and Cyclin D1^26^, P27, GSK-3, and so on (Fig. 8a)^29^. PTEN, both a protein and lipid phosphatase^29^, negatively regulates PI3K-Akt pathway through dephosphorylation of phosphatidylinositol (4,5)-phosphate (PIP2) and phosphatidylinositol (3,4,5)-phosphate (PIP3) at the D-3 position and prevents Akt activation^29^. PDK1, an upstream kinase whose activity is regulated by PI3K, always acts as a potential anticancer target because of its ability to phosphorlate and activate a diverse set of AGC kinase members (named after family members: PKs A, G and C), in particular the three Akt isoforms (PKBα/Akt1, PKBβ/Akt2 and PKBγ/Akt3)^27^,^58^. During the experiment, the treatments of EGCG or EGCG nanoparticles in MCF-7 cells resulted in significant (i) increase in the protein expression of Bax with concomitant decrease in Bcl-2 in favor of apoptosis; (iii) increase in the protein expression of p21 with concomitant decrease in Cyclin D1 in favor of cell cycle arrest; (iii) mutational inactivation of Akt and PDK1; (iv) overexpression of PTEN. Liang *et al*. have demonstrated that 30 μM EGCG blocked cell cycle progression at G1 phase in asynchronous MCF-7 cells^26^. However, whether G1 phase arrest of the cell cycle still exists when treated with EGCG nanoparticles in our experiments awaits further study.

In conclusion, EGCG nanoparticles, namely FA-NPS-PEG and FA-PEG-NPS, have been successfully synthesized and modified by FA and PEG, and showed better effectiveness than EGCG with respect to cellular uptake, the inhibition effect on proliferation of MCF-7 cells and modification of the expression of several key regulatory proteins in PI3K-Akt pathway, indicating that EGCG nanoparticles have successfully enhanced the targeting ability of EGCG *in vitro*. Besides, whether EGCG nanoparticles could improve the targeting ability and bioavailability of EGCG *in vivo* can’t be confirmed in this study and is still being investigated in our laboratory.Taking together with previous studies, our results again confirmed that EGCG inhibited on proliferation of MCF-7 cells with multiple functionalities. Compared to other anti-cancer drugs, EGCG as a natural component derived from tea possesses advantages of low toxicity, less cost, aqueous solubility, easy accessibility and daily consumption. However, whether EGCG or EGCG nanoparticles might be truly applied to the management of patients with cancer as cancer chemopreventive agents is still needed to undergo a very long process.

## Methods

### Materials

EGCG (≥95%) was purchased from Chengdu Purifa Science and Technology Development Co., Ltd. (Chengdu, China). 200 cps CS with DD of 95% and molecular weight (Mw) of 100 kDa derived from crab shell were obtained from Zhejiang Aoxing Biotechnology Co., Ltd. (Zhejiang, China). mPEG-SPA (2 000 Da) and NH_2_-PEG-COOH were purchased from Jiaxing Bomei Biotechnology Co. Ltd. (Zhejiang, China). EDC·HCl. DCC, NHS, TPP, FA, were purchased from Aladdin Industrial Corporation (Shanghai, China). Human breast cancer MCF-7 cells and trypsin/EDTA were purchased from the American Type Culture Collection (ATCC, Manassas, USA). Rhodamine B isothiocyanate and DMSO were purchased from Sigma Chemical Co. (Shanghai, China). Dulbecco’s modified eagle medium (DMEM), fetal bovine serum (FBS), Hank’s balanced salt solution (HBSS), penicillin, Hepes, insulin, streptomycin, gentamicin, 4′,6-diamidino-2-phenylindole (DAPI) and Hoechst 33342 were purchased from Dingguo Biotechnology Co. Ltd. (Chongqing, China).

### Preparation of NPS

The preparation of NPS followed the previous method with slight modifications^11^. Briefly, 100 mg of CS flakes were dissolved in acetic acid solution (0.175%, v/v) with constant stirring until the solution became clarified. CS solution was filtered through 5 μm filter (Millipore, USA) before mingled with 125 mg of EGCG. Then, under magnetic stirring (85-2A, Jintan, China), NPS were produced by the drop addition of 25 mL of 0.1% TPP solution to CS-EGCG solution. The mixture was stirred at room temperature for further 4 h to obtain NPS.

### Synthesis of FA grafted NPS (FA-NPS)

FA was conjugated to NPS through a method reported previously with modifications. 25 mg of FA was dissolved in 5 mL of 1/15 M potassium phosphate (pH 7.4), then 18 mL of NPS solution was mingled with 1 mL of FA solution, then 25 mg of EDC·HCl was added, and the mixture was allowed to stir at room temperature. After 1 h of stirring, the synthesized product, namely FA-NPS, was purified by dialysis against deionized (DI) water using a dialysis bag with 10 000 MWCO (Solarbio, Beijing, China) for 48 h to remove excess FA, followed by freeze drying.

### Synthesis of PEG grafted NPS (PEG-NPS)

PEG-NPS was synthesized according to the method described elsewhere with modifications^21^. 18 mL of EGCG-NPS was mixed with 50 mg of mPEG-SPA (2 000 Da), and stirred at room temperature for 4 h. Then the synthesized product was purified by dialysis against DI water using a dialysis bag with 10 000 MWCO for 48 h to remove excess mPEG-SPA, followed by freeze-drying.

### Synthesis of FA-NPS-PEG

FA-NPS-PEG was prepared by mingling 9 mL of unpurified FA-NPS solution with 25 mg of mPEG-SPA (2 000 Da), and stirring at room temperature for 4 h. Then the synthesized product was purified by dialysis against DI water using a dialysis bag with 10 000 MWCO for 48 h to remove excess mPEG-SPA and FA, followed by freeze-drying.

### Preparation of FA-PEG-NPS

As depicted in Fig. 1c, CS were modified by FA and PEG firstly^15^,^30^, then nanoparticles loaded with EGCG, named FA-PEG-NPS, were prepared by ionotropic gelation of CS with TPP^11^.

NHS ester of FA (FA-NHS (γ)) was prepared by activating the –COOH group using carbodiimide chemistry in accordance to the previous report^21^,^30^. DCC (93 mg) and NHS (77 mg) were dissolved into 12 mL of DMSO, then 0.3 g of FA was added. The mixture was stirred under room temperature and in the dark for 6 h, then the side product dicyclohexylurea (DCU) precipitated was removed by filtration. The filtrate was added into 100 mL of the mixture of diethyl ether and acetone with a volume ratio of 3:7, producing yellow precipitate immediately. Then the yellow product precipitated was collected for further use.

FA-PEG-COOH was synthesized by a procedure reported previously with modifications^21^. In brief, 0.45 g of NH_2_-PEG-COOH (3350 Da) were dissolved in 100 mL of pyridine, then the obtained FA-NHS (γ) were added and stirred at room temperature in the dark overnight. After evaporated under vacuum, the synthesized product was dissolved into 20 mL of DI water, and then 1 mL of the solution was transferred to eppendorf tube (1.5 mL) and centrifuged at 12 000 rpm for 30 min at room temperature. The supernatant was removed and dialyzed against 1/15 M potassium phosphate (pH 7.4) using a dialysis bag with 10 000 MWCO for 24 h and then dialyzed against DI water for 48 h, followed by freeze-drying.

FA-PEG-CS was synthesized by a procedure reported previously with modifications^21^. Briefly, 200 mg of CS was dissolved in 20 mL of 0.175% acetic acid solution and stirred at room temperature. 2.5 mg of EDC·HCl and 5.8 mg of NHS were dissolved in 5 mL of anhydrous DMSO, then 40 mg of FA-PEG-COOH was added, and the reaction was performed for 4 h. Then the obtained product was dialyzed against DI water using a dialysis bag with 10 000 MWCO for 72 h, followed by freeze-drying.

FA-PEG-NPS were achieved by dissolving 30 mg of FA-PEG-CS and 25 mg of EGCG in 25 mL of 0.175% acetic acid solution. After 0.5 h of stirring, 5 mL of 1% TPP were added into the mixture and stirred for further 4 h at room temperature in the dark. Finally, the obtained product was freeze-dried.

### Characterization of EGCG Nanoparticles

The zeta potentials of EGCG nanoparticles were determined using Zetasizer Nano-ZS90 (Malvern instruments, England) on the basis of Dynamic light scattering DLS techniques. FTIR spectra of EGCG nanoparticles were measured over 4000–400 cm^−1^ with a resolution of 4 cm^−1^ on a PerkinElmer Spectrum 100 spectrometer (PerkinElmer, USA). ^1^H NMR spectra of FA-NPS-PEG and FA-PEG-CS were measured in D_2_O containing 0.175% C_2_D_4_O_2_ using a 300 MHz spectrometer (Bruker Avance DPX 300, Switzerland)^35^. The morphologies and particles sizes of EGCG nanoparticles were characterized through SEM (S-4800, Shimadzu, Tokyo, Japan), operating at an accelerating voltage of 30.0 kV.

In order to determine EEs of EGCG in EGCG nanoparticles, the suspensions of EGCG nanoparticles were centrifuged at 10 000 g for 30 min at 25 °C, and free EGCG in the supernatant was determined by HPLC (LC-20A, Shimadzu, Tokyo, Japan) according to the previous method^59^. The EE of nanoparticles was calculated as equation (1).


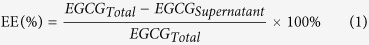


### Cell Culture

MCF-7 cells were maintained in DMEM supplemented with 50 units/mL penicillin, 10 mM Hepes, and 10 μg/mL insulin, 50 μg/mL streptomycin, 100 μg/mL gentamicin and 10% FBS under conditions of 5% CO_2_ and 95% humidity at 37 °C^60^. Cells used in this study were between passages 12 and 35.

### Cellular morphology

The effects of EGCG and EGCG nanoparticles on cellular morphology were conducted using Click-iT^TM^ EdU imaging kit (Ruibo Biotechnology, Guangzhou, China) according to the manufacturer’s protocol^42^. MCF-7 cells were seeded in a 96-well plate at 2.5 × 10^4^ cells/well and incubated for 6 h at 37 °C in humidified atmosphere containing 5% CO_2_. Then the old medium were replaced with fresh complete medium containing EGCG (200 μg/mL) or EGCG nanoparticles (containing 200 μg/mL EGCG), respectively, and cultured for another 48 h. Cells without EGCG or EGCG nanoparticles were taken as control. Then the medium were removed and 100 μL of 50 μM EdU in fresh complete medium was added into each wells. After 2 h incubation at 37 °C in humidified atmosphere containing 5% CO_2_, the cells were washed for 2 × 5 min with phosphate buffer saline (PBS) and then fixed with 4% paraformaldehyde in PBS for 30 min at room temperature. After removal of 4% paraformaldehyde, 2 mg/mL glycine was added and incubated for 5 min at room temperature. The wells were washed again with PBS and then permeabilized with 100 μL of 0.5% Triton X-100 in PBS for 10 min. After washed once more with PBS, the cells were incubated with 100 μL of 1 × Apollo^®^ Staining reaction buffer for 30 min at room temperature while protected from light. The cells were washed twice with 100 μL of permeabilization reagent and then washed for 2 × 5 min with methanol. For subsequent staining, the cells were washed once more with PBS and then incubated with 100 μL of Hoechst 33342 for 30 min at room temperature while protected from light. After washing three times with PBS, the morphology of the cells was observed using Inverted fluorescence microscope (Nikon, Japan).

### Evaluation of Cytotoxicity

Cytotoxicity was measured by using a methylene blue assay with modifications^61^,^62^. MCF-7 cells were seeded in a 96-well plate at 4 × 10^4^ cells/well and kept at 37 °C in humidified atmosphere containing 5% CO_2_ for 24 h. After removal of the medium and washed twice with sterile cold PBS, cells were treated with different concentrations of EGCG (0, 20, 40, 80, 160, 240, 320, 400 μg/mL) and EGCG nanoparticles (containing 0, 20, 40, 80, 160, 240, 320, 400 μg/mL EGCG) or control, respectively, and incubated for further 24 h. Then the medium was removed from each well, and washed cells with PBS again. 50 μL of methylene blue solution that contained 1.25% glutaraldehyde and 0.6% methylene blue in HBSS was added into each well to fix and stain cells. After 1 h incubation at 37 °C in humidified atmosphere containing 5% CO_2_, the methylene blue solution was removed from the wells. The 96-well plate was rinsed several times by immersion in Milli-Q water and dried. Each well was added 100 μL of elution solution (50% ethanol plus 49% PBS and 1% acetic acid), and the 96-well plate was incubated on a plate rotator at room temperature for 20 min. After 20 min incubation, the absorbance was measured at 570 nm using a MRX II Dynex plate reader (Dynex Technologies, Inc., Chantilly, VA, USA). Concentrations of pure compounds that decreased the absorbance by >10% when compared to the control were considered to be cytotoxic. At least three replications for each sample were used to determine the cytotoxic activity.

### Measurement of Cell Proliferation

The effects of EGCG nanoparticles on the growth of MCF-7 cells *in vitro* were measured using the methylene blue assay^61^. MCF-7 cells were maintained in DMEM supplemented with 10 mM Hepes, 10 μg/mL insulin, 50 units/mL penicillin, 50 μg/mL streptomycin, 100 μg/mL gentamicin, and 10% FBS at 37 °C in humidified atmosphere containing 5% CO_2_. MCF-7 cells were seeded at 2.5 × 10^4^ cells/well in a 96-well flat-bottom plate. After 6 h incubation, the old medium was removed and fresh medium containing EGCG (0, 20, 40, 80, 160, 240, 320, 400 μg/mL) or EGCG nanoparticles (containing 0, 20, 40, 80, 160, 240, 320, 400 μg/mL EGCG) was added, respectively, and then incubated at 37 °C in humidified atmosphere containing 5% CO_2_ for further 72 h. Cells without EGCG or EGCG nanoparticles were taken as control. Then the medium was removed from each well, and washed cells with PBS again. 50 μL of methylene blue solution that contained 1.25% glutaraldehyde and 0.6% methylene blue in HBSS was added into each well to fix and stain cells. After 1 h incubation at 37 °C in humidified atmosphere containing 5% CO_2_, the methylene blue solution was removed from the wells. The 96-well plate was rinsed several times by immersion in Milli-Q water and dried. Each well was added 100 μL of elution solution (50% ethanol plus 49% PBS and 1% acetic acid), and the 96-well plate was incubated on a plate rotator at room temperature for 20 min. After 20 min incubation, the absorbance was measured at 490 nm using a MRX II Dynex plate reader (Dynex Technologies, Inc., Chantilly, VA, USA). At least three replications for each sample were used to determine the cytotoxic activity.

### Cellular uptake studies

To study cellular uptake, five kinds of EGCG nanoparticles prepared above were mixed with 0.2 mL of 2 mg/mL Rhodamine B isothiocyanate in DMSO^35^,^43^, respectively, followed by incubation under room temperature and in the dark for 12 h. Then the mixture was dialyzed against DI water using a 10 000 MWCO dialysis bag for 72 h. The obtained product was centrifuged at 10 000 r/min for 10 min at 4 °C to obtain Rhodamine B-labeled EGCG nanoparticles. MCF-7 cells were seeded at 1 × 10^5^ cells/well in a 6-well plate equipped with pre-sterilized coverslip for each well and incubated for 12 h at 37 °C in humidified atmosphere containing 5% CO_2_. Then the old medium was replaced with fresh complete medium containing 1 mL of Rhodamine B-labeled EGCG nanoparticles (containing 200 μg/mL EGCG). The cells were cultured for further 24 h at 37 °C in humidified atmosphere containing 5% CO_2_, and then washed for 3 × 5 min with PBS. 500 μL of 4% paraformaldehyde in PBS were added into each well to fix the cells and incubated for 30 min at room temperature. Then 4% paraformaldehyde was removed and 500 μL of 2 mg/mL glycine was added and incubated for 5 min to remove the excess paraformaldehyde. The cells were washed again with PBS for 3 × 5 min and then permeabilized with 500 μL of 0.5% Triton X-100 in PBS for 10 min. After washing three times with PBS, the cells were treated with DAPI (20 μg/mL) and incubated for 30 min for nucleus staining. After removal of the media and washed twice with PBS, the cells were cover-slipped with Vectashield mounting media (Vector Laboratories Inc., Burlingame, CA, USA) and taken to a laser scanning confocal microscope (Nikon, Japan) for imaging^35^,^43^.

### Western blot analysis

MCF-7 cells were seeded in a 96-well plate at 2.5 × 10^4^ cells/well and incubated for 6 h at 37 °C in humidified atmosphere containing 5% CO_2_. Then the old medium was replaced with fresh complete medium supplemented with 240 μg/mL EGCG, FA-NPS-PEG or FA-PEG-NPS that containing 240 μg/mL EGCG, followed by incubation for further 48 h. Cells without EGCG or EGCG nanoparticles were taken as control. After removal of the medium and washed twice with PBS, the cells in each well were lysed with 40 μL of lysis buffer and then transferred to eppendorf tube (1.5 mL) using a cell scraper. After 30 min on ice, cell debris were removed through centrifugation (13 000 r/min, 20 min, 4 °C), and the supernatant containing proteins was collected and stored at −80 °C for later protein determination and gel electrophoresis.

The protein content in the supernatant was determined using the Pierce Micro BCA Protein Assay Kit (Thermo Fisher Scienfific, Rockford, IL, USA) according to the manufacturer’s instructions. After diluted with 5 × sample loading buffer, the cellular protein boiled for 20 min at 100 °C and then kept at −20 °C.

Our Western blotting protocols are essentially based on previous work^63^. 20 μL of cellular protein was size-fractionated by sodium dodecyl sulfate-polyacrylamide gel electrophoresis (SDS-PAGE, 12% PAGE), transferred onto PVDF membranes (Millipore, Billerica, MA, USA). Membranes were blocked with 5% non-fat dry milk in Tris-buffered saline Tween (TBST) for 1 h at 37 °C. After being blocked, the membranes were incubated overnight at 4 °C with primary antibodies at a dilution of 1:500~1:1000, which included mouse polyclonal anti-human p21, CyclinD1, *β*-actin, mouse polyclonal anti-human Bcl-2, Bax, goat polyclonal anti-human Akt1/2, *p*-Akt, rabbit polyclonal anti-human *p*-PDK1 and mouse monoclonal anti-human PTEN prepared in blocking buffer. After washed three times for 10 min with TBST, the membranes were incubated with horseradish peroxidase (HRP)-conjugated secondary antibodies (1:1000 dilution) for 1 h at 37 °C and then washed again. The membranes were exposed to ehanced chemiluminescence-plus reagents (ECL) from the Beyotime Institute of Biotechnology (Haimen, China) on Fusion Fx5 imaging system (Wuzhou Dongfang biotechnology, Beijing, China) according to the manufacturer’s protocol.

### Statistical Analysis

All data were presented as the mean ± SD of triplicate experiments. The statistical analysis was carried out with one-way analysis of variance (ANOVA) using the SPSS 19.0 software. The Fisher’s test significant difference (LSD) test was used for multi-group comparisons. A *p* < 0.05 was considered as statistically significant difference. The IC_50_ values were fitted by non-linear regression analysis using CalcuSyn Demo software.

## Additional Information

**How to cite this article**: Zeng, L. *et al*. Preparation and characterization of (−)-Epigallocatechin-3-gallate (EGCG)-loaded nanoparticles and their inhibitory effects on Human breast cancer MCF-7 cells. *Sci. Rep.* **7**, 45521; doi: 10.1038/srep45521 (2017).

**Publisher's note:** Springer Nature remains neutral with regard to jurisdictional claims in published maps and institutional affiliations.
